# Posterior Reversible Encephalopathy Syndrome in Pediatric Hematologic-Oncologic Disease: Literature Review and Case Presentation

**Published:** 2014

**Authors:** Mohammad Thaghi ARZANIAN, Bibi Shahin SHAMSIAN, Parvaneh KARIMZADEH, Mohammad KAJIYAZDI, Fatima MALEK, Mohammad HAMMOUD

**Affiliations:** 1 Pediatric Congenital Hematologic Disorders Research Center, Mofid Children Hospital, Shahid Beheshti University of Medical Sciences, Tehran, Iran; 2 Pediatric Neurologist, Mofid Children Hospital, Shahid Beheshti University of Medical Sciences, Tehran, Iran

**Keywords:** Posterior reversible encephalopaty syndrome(PRES), Hematologic disease, Leukemia, Cyclosporine

## Abstract

**Objective:**

Posterior reversible encephalopathy syndrome (PRES) is a cliniconeuroradiological disease entity, which is represented by characteristic magnetic resonance imaging (MRI) findings of subcortical/cortical

hyperintensity in T2-weighted sequences. It is more often seen in parietaloccipital lobes, and is accompanied by clinical neurological changes. PRES is a rare central nervous system (CNS) complication in patients with childhood hematologic-oncologic disese and shows very different neurological symptoms

between patients, ranging from numbness of extremities to generalized seizure. In this article, we will review PRES presentation in hematologic-oncologic patients. Then, we will present our patient, a 7-year-old boy with Evans syndrome on treatment with cyclosporine, mycophenolate mofetil (MMF) and prednisone, with seizure episodes and MRI finding in favour of PRES.

## Introduction

Neurological complications may happen during treatment of childhood hematologic malignancies and solid tumors, of which headache and seizures are the most common (1).

In 1996, Hinchey et al. described the clinical entity of reversible posterior leukoencephalopathy syndrome (1-3). It is also known as posterior reversible encephalopathy syndrome (PRES) or posterior reversible leukoencephalopathy syndrome. This syndrome was recognized since magnetic resonance imaging (MRI)

became available. Typical transient lesions on MRI (i.e., edema of the subcortical white matter shown on T2 weighted images and fluid attenuation inversion recovery (FLAIR) images) are pathognomonic of the syndrome. In addition, PRES can affect basal ganglia, cerebellar hemispheres, and brainstem (1). The main clinical features of this syndrome are headaches, seizures, altered levels of mental status, and cortical blindness (1). These clinical and radiographic perturbations are transient, and patient’s outcome is usually favorable (1-3). 

PRES has been initially described in adult patients with eclampsia, renal dysfunction, and systemic lupus erythematosus (SLE), and a robust association with severe hypertension and immunosuppression was described. Also, PRES is a clinicoradiological phenomenon, which is associated with various medical conditions in children with hematologic and malignant disease including leukemia, solid tumors, aplastic anemia, and autoimmune disease. Understanding of this syndrome and its sequelae in this population is limited by the small number of reported patients and by an incomplete understanding of the disorder itself (1, 3, 4-6).

In this article, we will review literature about PRES, all well-documented PRES cases during childhood

cancer and hematologic disorders, and then we present a 7-year-old boy with Evans syndrome who developed PRES during the immunosuppressive treatment.

**PRES Epidemiology**

The global incidence of PRES is unknown. The only epidemiological data are from retrospective studies

of cases seen between 1988 and 2008. PRES has been reported in patients aged 4 to 90 years. There is

a significant female predominance which may reflect some of the causes. Many patients with PRES have

comorbidities that may be severe conditions, e.g., bone marrow or solid organ transplantation, chronic renal failure, and chronic hypertension (7).

**Pathophysiology**

The pathophysiology of PRES has been remained controversial until now. The two main hypotheses

contradict each other. One is impaired cerebral autoregulation that is responsible for an increase in cerebral blood flow (CBF), while another is endothelial dysfunction with cerebral hypoperfusion. This hypoperfusion hypothesis may be most related to cases of PRES associated with cytotoxic therapy. In both hypotheses, the outcome of the cerebral blood perfusion abnormalities is blood brain barrier dysfunction with cerebral vasogenic edema. Onder et al. detected hypertensive crisis as the most common trigger of PRES in 59%. PRES related to hypertension might be due to sudden increase in blood pressure causing disruption of the autoregulatory mechanisms of the CNS vasculature, which causes vasoconstriction and vasodilatation, and breakdown of the bloodbrain barrier. The immunosuppressive and cytotoxic drugs may cause and aggravate hypertension and may decrease seizure threshold. ntrathecal chemotherapy may cause cerebral vasospasm, which contributes to cerebral vascular autoregulation impairment. Also, it appears that renal dysfunction predisposes to PRES, because of chronic uremia or fluid overload. One of the most important immunosuppressive drugs that can induce PRES is cyclosporine. The distribution of T2 abnormalities in cyclosporine-induced PRES can be related to the sparse sympathetic innervation in the posterior circulation. Pathologic studies have revealed that the relative density of sympathetic innervations is greatest in the internal carotid and anterior cerebral territories and least in the basilar artery and its branches. Hence, breakdown of autoregulatory mechanisms would first occur in the more poorly innervated vessels of the posterior circulation (1,6,7-15).

**Conditions most commonly associated with PRES:**

**Toxic Agents:**

**Cytotoxic agents: **Alkyl ating agents

Cisplatin, Oxaliplatin, Carboplatin

Anti-metabolites: Gemcitabine, Cytarabine, Methotrexate

Mitotic inhibitors: Vincristine

Irinotecan

Others: L-asparaginase

Anti-angiogenic agents: Bevacizumab, Sunitinib

Immunomodulatory cytokines: Interferon-alpha,

Interleukin-2

Monoclonal antibodies: Rituximab (anti-CD20),

Infliximab (anti-TNF)

Intravenous immunoglobulin (IVIG)

Anti-TNF-protein: Etanercept

Anti-lymphocyte globulin

**Immunosuppressive agents:**

Anticalcineurin agents: Cyclosporine A, Tacrolimus

(FK 506),

Sirolimus

High-dose corticosteroid therapy (e.g., dexamethasone and methylprednisolone)

**Other agents: **Linezolid, Granulocyte-stimulating factor, Erythropoietin

Intravenous contrast agents

**Blood transfusion**

**Hypertension, Infection/Sepsis/Septic Shock**

**Preeclampsia/Eclampsia**

**Autoimmune disease**

**Transplant**

Stem cell transplantation, solid organ

**Other Conditions**

Sickle cell disease, Guillain-Barré syndrome, hypomagnesaemia, hypercalcemia, tumor lysis syndrome, porphyria, pheochromocytoma, and Cushing syndrome (7).

**Clinical Manifestations of PRES**

The typical features of PRES include consciousness impairment, seizure activity, headaches, visual

abnormalities, nausea/vomiting, and focal neurological deficit (7). Consciousness impairment may range in severity from confusion, somnolence, and lethargy to encephalopathy or coma. All these clinical features do not describe in all pediatric PRES patients. In a study on 25 children with PRES, 44% manifested all four clinical symptoms, 32% demonstrated three, 16% showed two, and 8% had only one symptom. Kwon et al. reported 12 patients that presented with seizures (42%), visual disturbances (33%), headache (17%), or altered mental status (8%). Incecik et al. detected the most common clinical features as seizure (6/9), headache (6/9), and altered consciousness (4/9). The other symptoms were nausea and vomiting and blurred vision (7,12). 

**Diagnosis**

PRES is a clinico-radiological entity. The intensity and severity of its clinical manifestations are different

and may need ICU admission. Imaging findings also vary in severity. Complete familiarity with the imaging criteria is essential for the diagnosis. Suggestive clinical manifestations together with radiological criteria established the diagnosis of PRES. In suspicious cases, the clinical and radiological improvement that occurs once appropriate treatment is given, confirms the diagnosis. However, there are no consensual guidelines for validation of PRES diagnosis (1, 2, 7).

**Roles for computed tomography and MRI in the diagnosis of PRES**

Computed tomography (CT) findings are often normal or nonspecific. Hypodensities in a suggestive

topographic distribution suggest PRES. Cerebral MRI is the important investigation for the diagnosis of PRES. Proton-density and T2-weighted images show regions of high signal indicating edema. Fluid-attenuated inversion recovery (FLAIR) sequences also visualize the lesions. The application of FLAIR has been shown to improve the diagnosis of PRES and the detection of subcortical and cortical lesions in PRES. T1-weighted images demonstrate low-intensity foci. Diffusion-weighted imaging (DWI) is normal but the apparent diffusion coefficient is enhanced. Finally, enhancement is seen in approximately half the cases. MRI is more preferable than CT for the diagnosis of PRES (7).

**Fig 1:**
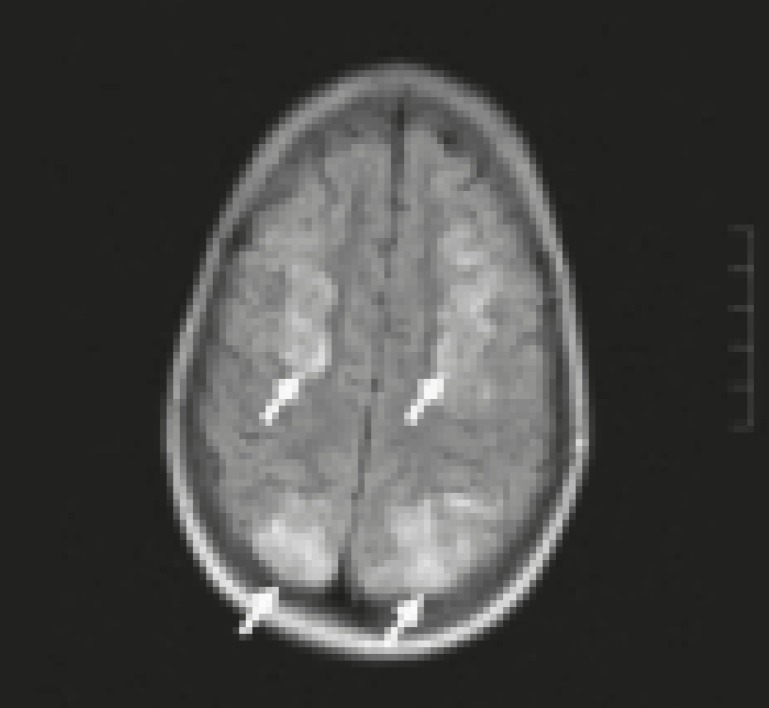
MRI in patient with PRES: Typical hyperintense spots at cortical-subcortical level in T2-weighted

sequences in parietal and occipital regions (white arrows)

**Fig 2:**
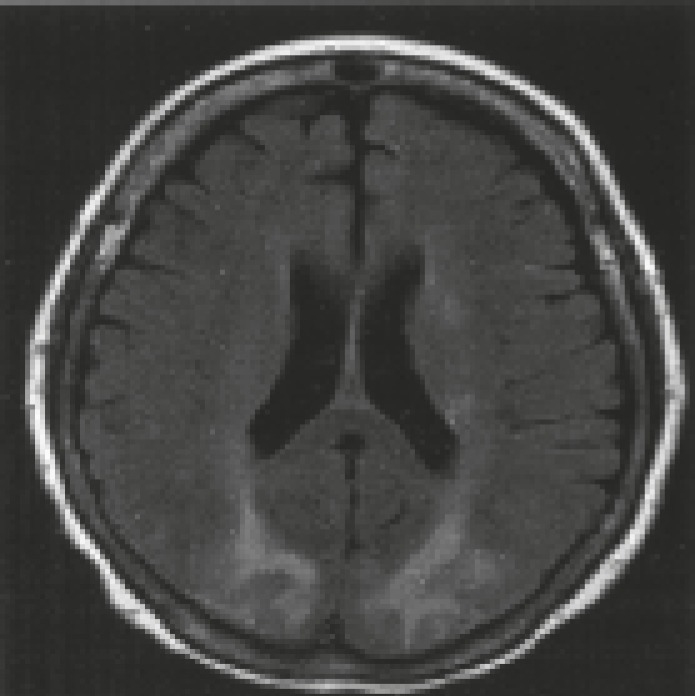
FLAIR MRI demonstrating bilateral zones of subcortical white matter hypersignal in the posterior frontal and occipital lobes

**Fig 3:**
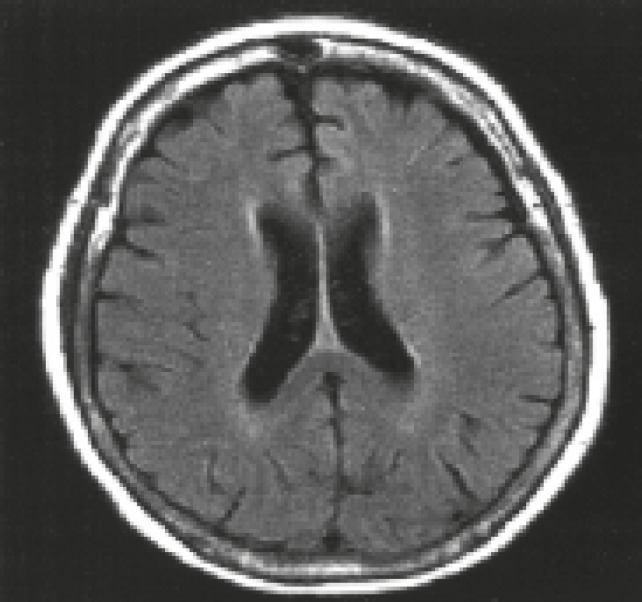
FLAIR MRI demonstrating resolution of hypersignal zones

**Fig 4:**
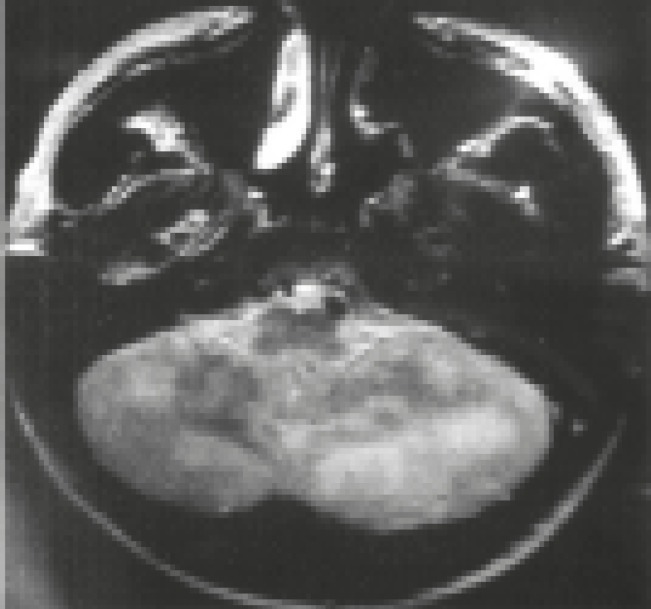
T2-weighted MRI of the brain demonstrating foci of high signal in the cerebellum

**Fig 5:**
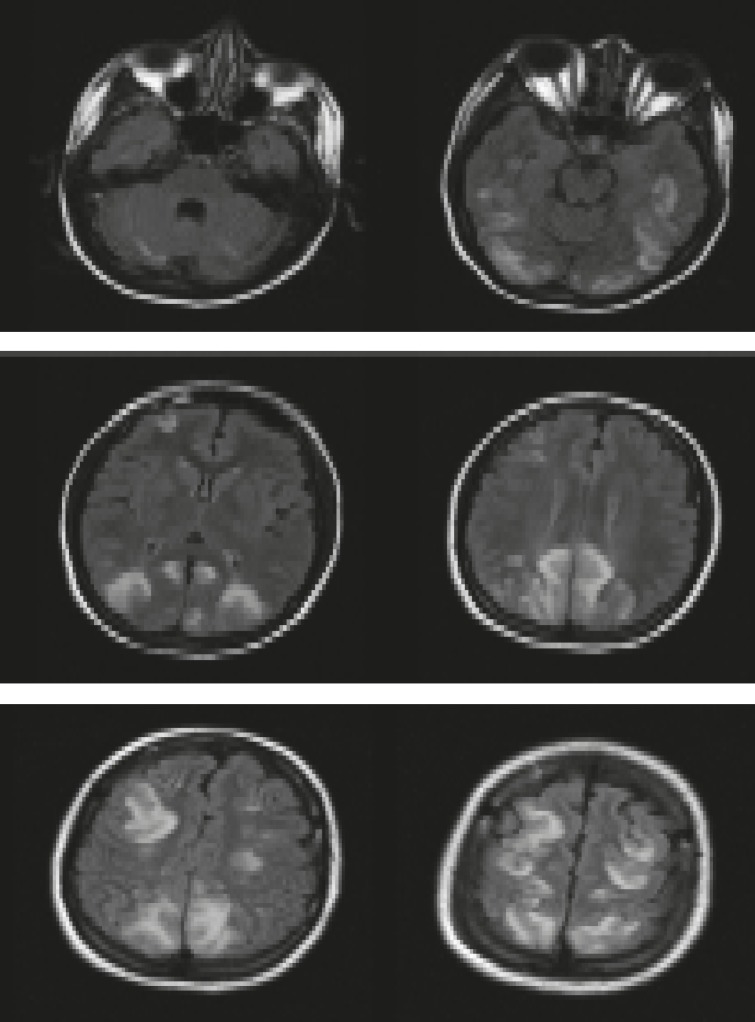
Cerebral MRI in a patient with PRES

Fluid-attenuated inversion recovery (FLAIR) sequence showing bilateral high-signal foci in the cerebellum, basal ganglia, and occipital, parietal, frontal, and temporal lobes (1-4,7).

**Radiological Characteristics of PRES (** 7 **)**

Until recently, It was believed that PRES consistently produce bilateral and symmetric regions of edema

typically located in the white matter and predominating in the posterior parietal and occipital lobes. Sometimes, edema has been described in the frontal lobes, temporal, basal ganglia or cerebellum and brainstem in the posterior fossa, and cortical gray matter. 

**The four radiological patterns of PRES are as below:**

**Fig 6:**
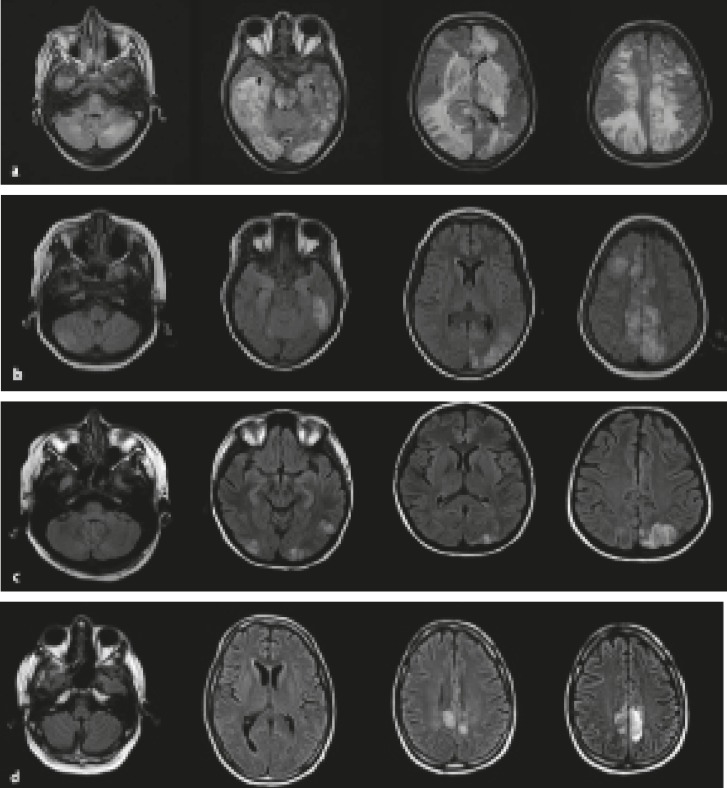
Four main MRI patterns of PRES

**a. Holohemispheric watershed pattern (23%) **(Fig.6a). A swath of confluent vasogenic edema extends through the frontal, parietal, and occipital lobes. Involvement of the temporal lobes is less noticeable This topography matches the watershed zone between the anterior and posterior cerebral arteries, on the one hand, and the middle cerebral artery, on the other.

**b. Superior frontal sulcus pattern (27%) **(Fig.6b). Patchy edema predominates in the frontal lobes along the superior frontal sulci. The parietal and occipital lobes are involved variably.

**c. Dominant parietal-occipital pattern (22%) **(Fig.6c). In this pattern that was previously thought to be typical of PRES, the posterior part of the parietal and occipital lobes is predominantly involved. The edema alters in severity from mild to extensive.

**d. Partial or asymmetric expression of the primary patterns (28%) **(Fig.6d). The partial form is

described as bilateral absence of edema in either the parietal or the occipital lobes. The frontal lobes are often involved. The asymmetric form is characterized by unilateral absence of edema in either a parietal or an occipital lobe. Finally, in the partial and asymmetric form, there is both absence of involvement of either the parietal or the occipital lobes and asymmetric abnormalities in the affected parietal or occipital lobes.

**Complications diagnosed radiologcally at presentation of PRES:**

Complications diagnosed radiologically at presentation of PRES are cerebral ischemia and cerebral hemorrhage (7).

**Differential diagnosis:**

The non - specific clinical manifestations and multiplicity of radiological patterns increase diagnostic 

challenges. Various conditions may resemble PRES, including ictal or post-ictal state (with or without status epilepticus), infectious encephalitisprogressive multifocal leukoencephalopathy (PML), acute disseminated encephalomyelitis, mitochondrial myopathy encephalopathy lactic acidosis and strokelike

episodes syndrome (MELAS), Creutzfeldt-Jakob disease, vasculitis, cerebral venous sinus thrombosis,

and ischemic stroke (watershed or posterior cerebral artery territory). The MRI characteristics of these conditions are helpful in diagnosis (7).

**Treatment**

The treatment strategy relates general measures to correction of the underlying cause of PRES. An early

etiologic diagnosis permits immediate correction of the cause of PRES. Patients may need blood pressure

control, withdrawal of cancer chemotherapy or immunosuppressive agents, cesarean section, dialysis, or other interventions. Immediate correction of the cause is essential to decrease the risk of ischemia or bleeding and therefore to prevent permanent disability or death. In patients with seizure, anticonvulsant therapy is recommended even after a single seizure. Conversion of anti-seizure therapy to non-hepatic microsomal enzyme-inducing drugs is recommended as soon as the clinical condition of the patient stabilizes. Morris et al. recommended ceasing the anticonvulsant therapy after 3-6 months in uncomplicated cases. They also recommended an anticonvulsant regimen for at least 12 months after the following seizure episode in patients with abnormal ﬁndings on electroencephalogram or cranial MRI and recurrent seizures. Lucchini et al. advised anticonvulsant therapy for 12 months in patients with brain damage. No consensus has yet been achieved about which patients should receive antiepileptic drugs for seizures after PRES (1-4, 6,7).

**PRES in hematologic-cancer patients**

Pediatric patients with hematologic–cancer disease are at risk of PRES. Understanding of this syndrome and its sequelae in this population is limited by the small number of reported patients and by an incomplete understanding of the disorder itself. A variety of factors have been defined in the etiology of PRES. The most common factors in this group of patients are immunosuppressive drugs (cyclosporine, anti-thymocyte globulin, rituximab, tacrolimus, interferon) and chemotherapeutic agents (methotrexate, L-asparaginase, adriamycin, cyclophosphamide, cytosine arabinoside, vincristine). Also, sickle cell disease, hypertension, acute blood pressure changes, renal failure, tumor lysis syndrome, infection, sepsis, shock, andtransplantation (stem cell and organ) are some factors that can cause PRES in this group of patients (8).

Throughout the treatment of acute childhood leukemia, PRES may occur as a complication. Induction chemotherapy regimens of acute lymphoblastic leukemia (consisting of systemic steroids, repetitive intrathecal methotrexate, vincristine, and L-asparaginase) may comprise predisposing factors of PRES.

Asparaginase-induced toxicity or methotrexate-induced encephalopathy should be differentiated from PRES developing as a complication during the acute leukemia treatment. Methotrexate-induced encephalopathy shows a tendency to involve the cerebral white matter. In addition, methotrexate-induced encephalopathy has a tendency to occur in a certain time period, usually a few weeks after intrathecal methotrexate infusion. Moreover, methotrexate-induced encephalopathy usually involves no restriction of diffusion on diffusion MRI. These characteristics are helpful in the differential diagnosis of chemotherapy-induced leukoencephalopathy and PRES (4).

A few cases of PRES related to hematopoietic stem cell transplantation (HSCT) in children have been reported. Early recognition of PRES and proper management are required to decrease the risk of permanent neurological disability. In cases of PRES developing as a complication after hematopoietic stem cell transplantation, immunosuppressive agents and hypertension appear to be the main predisposing factors for PRES. Tacrolimus and cyclosporine have been considered as predisposing factors in PRES. However, the onset of PRES could not be associated a certain type of drug, because multiple immunosuppressive agents, including tacrolimus, cyclosporine, mycophenolate mofetil, and systemic steroids were administered simultaneously in various combination (4). Heo described a girl who received an HLA- identical sibling bone marrow transplant for myelodysplastic syndrome with PRES. Endo reported 4 patients with PRES, which 2 of them had hematologic disease. One of them was a case of severe aplastic anemia who developed PRES on day 22 after SCT. She was on cyclosporine A for Graft-versus-host disease (GVHD) prophylaxis. After 5 years of follow-up, she was complicated with intractable seizure and mental retardation (5).

A retrospective chart review by Kim et al. was performed on 19 patients aged less than 18 years who developed PRES as a complication during treatment of acute childhood leukemia. PRES was most often observed during acute lymphoblastic leukemia induction chemotherapy (n ¼ 9, 47.4%) and after hematopoietic stem cell transplantation (n ¼ 8, 42.1%). Nine (47.4%) patients developed the complication of PRES during acute lymphoblastic leukemia induction chemotherapy. The other two patients developed the complication of PRES during prophylactic intracranial irradiation therapy and maintenance chemotherapy. Among 8 patients with the complication of PRES after hematopoietic stem cell transplantation, 5 (62.5%) had a history of hypertension. In contrast, among 11 patients with the complication of PRES without hematopoietic stem cell transplantation, only one (9.1%) had a history of hypertension. Moreover, unlike other leukemia induction chemotherapy, PRES developed only in patients who received acute lymphoblastic leukemia induction chemotherapy. PRES patients needed long-term anticonvulsant therapy (n ¼ 9, 50.0%) and manifested intractable seizures (n ¼ 3, 16.7%). Sequelae were evident in long-term follow-up MRIs (n ¼ 5, 26.3%). Therefore, it seems that acute lymphoblastic leukemia chemotherapy regimens comprised the main predisposing factors for PRES complicated during induction chemotherapy, compared to hypertension and immunosuppressive agents after hematopoietic stem cell transplantation (4).

Incecik et al. reported 9 cases of PRES (7 boys and 2 girls) with mean age of 7.78±3.76 years (ranging 3-13 years). The main etiologies were as follows: taking intrathecal methotrexate for acute lymphoblastic leukaemia (ALL-L2) in two patients; taking cyclosporine for allogeneic bone marrow transplantation for thalassemia in another two patients; taking intrathecal cytarabine (Ara-C) for acute myeloblastic leukemia (AML) in one patient; taking cyclophasphamide for nonHodgkin’s lymphoma in one patient, taking IVIG for guillain-barré syndrome (GBS) in one patient; and the last two patients had acute hypertensive crisis and chronic renal failure. Importantly, 7 children were normotensive and were not hypertensive at presentation and follow-up. PRES resolved completely in all of the patients (12). Morris et al. reported 11 patients (7 females) who had radiological and clinical features consistent with PRES and were under treatment for cancer at St. Jude Children’s Research Hospital between January 1995 and January 2005. The average age of them at the time of PRES onset was 10.4 years. Primary diagnoses were acute leukemia (n=8), non-Hodgkin lymphoma (n=2), and Ewing sarcoma (n=1). All patients had previously taken chemotherapy, and 8 patients were receiving remission induction chemotherapy at the time of PRES onset. One patient (patient 4) had undergone bone marrow transplantation 2 months before PRES onset and was taking tacrolimus for treatment of chronic GVHD. Six patients were taking steroid therapy at the time PRES began, and 6 patients had previously received methotrexate. PRES occurred in 8 patients in the induction phase of treatment, and all 11 patients had hypertension (5 chronically). Seizure activity was proximate to cytarabine and tacrolimus administration in three patients and further seizures occurred in two patients when these medications were re-administrated. Coagulation and chemistry studies were normal. The white blood cell counts of all patients were normal during PRES. Calcium and magnesium levels were also normal in all patients. Acute EEG studies of five patients were completed; the detected abnormalities included nonconvulsive focal status epilepticus (which responded to intravenous midazolam), epileptiform activity, and background slowing. Concurrent brain MRI demonstrated T2 signal abnormalities in all 11 patients, restricted diffusion in 4, and hemorrhage in 3. Follow-up MRI demonstrated chronic changes consistent with a previous hemorrhage in three and evidence of prior parenchymal ischemia in one. Three patients developed epilepsy and chronic anticonvulsant therapy was continued for them. The results of this study showed that PRES is an increasingly recognized complication of pediatric cancer treatment. Risk factors for PRES in pediatric cancer patients were hypertension (not necessarily acute) and remission induction chemotherapy. Common side effects of cancer treatment, such as hypertension, systemic inflammatory response, sepsis, coagulopathy, hyperviscosity syndrome, and electrolyte derangement, were presumed to negatively affect outcome. Most of the patients developed PRES during the induction phase of treatment. The reason for this timing has not yet been clear. It is likely that unidentified systemic factors associated with the induction phase of treatment predispose patients to PRES. Endothelial dysfunction with subsequent blood–brain barrier disruption is a proposed mechanism of PRES and may occur with induction treatment uses repetitive systemic and intrathecal administration of chemotherapy. Endothelial dysfunction may also account for the increased incidence of hemorrhage in this group of patients being treated for AML. MR images often show atypical findings, some of which are irreversible. A significant number of patients develop epilepsy despite clinical and radiographic evidence of recovery. Won reported 8 patients with PRES; 4 of them had acute lymphocytic leukemia, 1 had aplastic anemia, and 3 had solid tumors (1 patient each for neuroblastoma, Ewing sarcoma, and osteosarcoma). Allogeneic stem cell transplantation was done in 2 patients. Immunosuppressive agents, such as tacrolimus and cyclosporine A were used in 3 patients. One neuroblastoma patient was in immediate postoperative status. All patients experienced seizure attacks of different types and showed typical MRI findings.

Follow-up MRIs revealed significant improvements. From this review, chemotherapy and surgery might be considered as additive causes for PRES other than immunosuppressive agents. Thus, careful examination of the patients receiving chemotherapy and surgery was required to find out this uncommon but good prognostic complication (16).

Lucchini described that encephalopathy syndrome in children with hemato-oncological disorders is not always posterior and reversible. He presented 12 cases of PRES in children who underwent intensive chemotherapy regimens. All patients survived the acute event which showed a clinical recovery of the acute neurological signs in 1–3 days and normalization of the pattern of EEG in a period ranging from 1 to 8 months. 

During long-term follow-up, 4 patients developed either clinical impairment or EEG-MRI anomalies.

He suggested that a long-term follow-up is required to determine the reversibility of the neurological events. Clinical observation, as well as EEG and MRI should be considered in follow-up evaluations. Siebert reported 18 pediatric patients with PRES with mean age 9 years (IQR 7-12). Most common predisposing causes were renal and haemato-oncologic diseases frequently associated with endotheliotoxic cytostatic medication. After 5 years of Follow-up, she had complicated intractable epilepsy and mental retardation. Frontal lesions occurred as commonly as parietal lesions followed by occipital lesions. The superior frontal sulcus topographic lesion pattern occurred as frequently as the parietal-occipital one. In 38% of cases, residual lesions were encountered with focal laminar necroses which are most frequent. Initial clinical syndromes associated with PRES were: 

seizures in 18, altered mental state in 5, and hemiparesis and visual disturbances in 2 children. Mean arterial blood pressure at the onset of PRES was 140/85 mmHg (IQR systolic: 124-169, diastolic: 78-93 mmHg). He concluded that pediatric PRES in this cohort comprises a broad radiological and clinical spectrum. The occurrence of frontal lesions, a superior frontal sulcus associated lesion pattern, and the development of focal laminar necrosis seem to be common in children (3).

So, considering of PRES in hematologic-oncologic patients with neurologic symptoms including seizures episodes is advised, and evaluation by MRI images, especially FLAIR sequences has the most accurate diagnostic value.

**Case presentation**

The patient is a 7-year old boy with a history of thrombocytopenia, then progression to direct and indirect coombs positive immune hemolytic anemia. Based on evaluation and diagnosis of Evans syndrome during the course of disease, he received different immunosuppressive treatments, including corticosteroids (prednisone and pulse therapy of methylprednisolone), cyclosporine, IVIG, and blood transfusion. He was refractory to treatment and had been dependent on blood transfusion. He was on blood transfusion every other day. On May 2012, 4 months after the onset of the disorder, he was referred to our Hematology Department. At the time of presentation, he had cushingoid face, pallor (RBC:1.890000/mm3, Hb: 5.4 gr/dl, MCV: 86 , Platelet: 129000/mm3, Reticulocyte: 10%), jaundice (BilT: 7.8 mg/dl, BilD: 2 mg/dl) and splenomegaly (169 mm). Complementary evaluations, including virology tests and collagen vascular tests were negative. Direct and indirect coombs tests as well as antibody screening tests were positive for both IgG and C3d. Bone marrow aspiration revealed erythroid hyperplasia and ow cytometry was normal. We started treatment with rituximab 375 mg/m2/week for 4 weeks and cyclosporine 7 mg/kg/day in divided doses (Q8h) in addition to corticosteroid. After 4 courses of rituximab, the patient showed a very good response to the treatment. Anemia and icterus improved and blood transfusion was stopped. So, in this phase, tapering of corticosteroids was started and mycophenolate mofetil (MMF) was added to the treatment. The patient was on follow-up and he was clinically stable. 

Two months later during follow up and treatment with cyclosporine, MMF, and tapered prednisone, he presented to emergency department with seizure episodes without fever. His blood pressure was 140/90. His CBC was as follows: WBC 2700/mm3, Hb 11.3 gr/ dl, platelet 68000/mm3, reticulocyte count 4.5%. PT, PTT, and biochemistry tests including BS, Ca, and Mg were normal. He was admitted to PICU. Anticonvulsant therapy, including diazepam, phenytoin, phenobarbital, and supportive therapy was started. Brain CT and MRI, MRV, and MRA were performed. Lesions were in favour of PRES in occipital area. EEG was normal. Based on diagnosis of PRES, cyclosporine was discontinued. Tapering and stopping of MMF were started and then treatment was changed to azathioprine. One month later, brain MRI was performed and was completely normal. He was clinically stable and treatment with azathioprine, low dose corticosteroid, and phenobarbital was continued. Because of history of long-term treatment with corticosteroids, bone mineral densitometry and endocrine consultation were done, and the patient received pamidronate. 

He was on follow-up until 2 months later, when he was admitted again because of severe back pain and low grade fever. He was not able to walk. Imaging evaluation including lumbosacral MRI revealed spondylodiscitis and collapse of L2-L3 vertebrae. Based on history of immunosuppressive therapy and fever, antibiotic treatment was started and consultations with orthopedic and neurosurgeon teams were done. First, biopsy sampling with CT guide was done, but the result of sampling was not conclusive and revealed just an inflammatory processes. Use of a lumbosacral cast was not effective. During admission, the patient developed high persistent fever and pancytopenia. At this time, complete evaluation was done. The results of blood cultures, PCR assay for mycobacterium tuberculosis, and virology tests, including Parvovirus B19, HIV, HbsAg, HbsAb, CMV, and EBV were negative. Bone marrow aspiration showed severe hypo-cellular marrow. He received IVIG and GCSF, in addition to wide spectrum antibiotic therapy. Because of persistent pancytopenia and fever, imaging for evaluation of fungal infection including chest CT was done. The CT report was suggestive of fungal infection. The result of galactomannan test was negative. Treatment with liposomal amphotericin B was started, but because of the persistent fever and no response to liposomal amphotericin B, voriconazole was added to the treatment.

The patient had a neurosurgical intervention because of progression of paresthesia and paraplegia. Open surgery and tissue sampling from the site of lumbar lesions showed fungal invasion of bone (Aspergillosis). Tissue sampling for malignancy was negative. Also, PCR assay for Mycobacterium tuberculosis was negative. 

Unfortunately, treatment with combination therapy of antifungal drugs was not effective and the patient died 1 year after the onset of disease on March 2013.

**In conclusion, **PRES is a clinico-neuroradiological disease entity representing with characteristic MRI findings of subcortical/cortical hyperintensity in T2-weighted sequences, more often observed in parietaloccipital lobes, accompanied by clinical neurologic alterations. PRES is a rare central nervous system complication in child hematologic-oncologic patients and presents with various neurological symptoms, ranging from numbness on extremities to a generalized seizure. As a result, careful examination of patients receiving chemotherapy, immunosuppressive therapy, and surgery was required to find out this uncommon but good prognostic complication.
